# Nematode genome announcement: A chromosome-scale genome assembly for the *Pristionchus pacificus* reference mapping strain PS1843

**DOI:** 10.2478/jofnem-2024-0063

**Published:** 2024-09-11

**Authors:** Waltraud Röseler, Ralf J. Sommer, Christian Rödelsperger

**Affiliations:** Department for Integrative Evolutionary Biology, Max Planck Institute for Biology, Max-Planck-Ring 9, 72076 Tübingen, Germany

**Keywords:** Comparative genomics, Evolution, Gene loss, Genome announcement, *Pristionchus pacificus*, Washington

## Abstract

*Pristionchus pacificus* is a free-living nematode that shares many features with *Caenorhabditis elegans*, such as its short generation time and hermaphroditism, but also exhibits novel traits, i.e., a mouth-form dimorphism that enables predation. The availability of various genetic tools and genomic resources make it a powerful model organism for comparative studies. Here, we present an updated genome of the *P. pacificus* strain PS1843 (Washington) that is most widely used for genetic analysis. Assembly of PacBio reads together with reference-guided scaffolding resulted in a chromosome-scale genome spanning 171Mb for the PS1843 strain. Whole genome alignments between the *P. pacificus* PS1843 genome and the genome of the *P. pacificus* reference strain PS312 (California) revealed megabase-sized regions on chromosomes III, IV, and X that explain the majority of genome size difference between both strains. The improved PS1843 genome will be useful for future forward genetic studies and evolutionary genomic comparisons at the intra-species level.

The free-living nematode *Pristionchus pacificus* is an established model species for comparative studies with *Caenorhabditis elegans* and for studying developmental plasticity (Sommer and McGaughran, 2013; Sommer et al., 2017). *P. pacificus* has an advanced genetic toolkit and a high quality genome (Rödelsperger et al., 2017; Han et al., 2020; Hiraga et al., 2021; Igreja et al., 2022). Several forward genetic studies have exploited the wealth of natural isolates in order to investigate the genetic basis of various traits (Mayer et al., 2015; McGaughran et al., 2016; Moreno et al., 2016; Dardiry et al., 2023). One particular strain of *P. pacificus* (PS1843) that had originally been isolated from the state of Washington has been frequently employed for genetic analysis (Srinivasan et al., 2002; Sieriebriennikov et al., 2020; Rillo-Bohn et al., 2021). Low coverage sequencing data for this strain has been available since the first sequencing of the *P. pacificus* genome and revealed more than a million differences with regard to the *P. pacificus* PS312 genome, i.e., sequence divergence ~1% (Dieterich et al., 2008; Rödelsperger et al., 2014). Recently, we resequenced and assembled the complete PS1843 genome from Illumina short read data (Prabh and Rödelsperger, 2022). Based on benchmarking of universally conserved single copy orthologs (BUSCO) (Simão et al., 2015), the completeness of the Illumina genome was comparable to the chromosome-scale genome of reference strain PS312 from California that was assembled from PacBio long reads (88.8% vs. 92.6%, Table 1). However, the genome was still quite fragmented (Table 1), and specific analysis of gene losses often turned out to be inconclusive due to the presence of assembly gaps (Prabh and Rödelsperger, 2022). Therefore, we decided to resequence the *P. pacificus* PS1843 genome using the PacBio platform. Worms were washed off of 100 plates using M9 buffer, and genomic DNA was extracted using the Qiagen genomic DNA extraction kit according to the manufacturer’s instructions. DNA quality and quantity were determined with a NanoDrop spectrometer (ThermoFisher), a Qubit fluorometer (Invitrogen), and a Femto pulse system (Agilent). A genomic library was prepared according to the manufacturer’s protocol (Pacific Biosciences) using a BluePippin size-selection system and was sequenced on a multiplexed run of a PacBio Sequel II SMRT cell. This yielded around 730,000 reads with a median read length of 11.6kb (Interquartile range: 4.5–24.4kb). This translates into 11.7Gb of raw sequence, which corresponds to around 70x coverage of the *P. pacificus* genome. Raw PacBio reads were assembled by the software Canu (version 1.4, options: genomeSize=180m -pacbio- raw) (Koren et al., 2017). This resulted in an initial assembly of 63 contigs spanning 171 Mb with an N50 value of 5.9 Mb. BLASTN searches (e-value < 10^−5^) did not identify any of the contigs as contamination from the *Escherichia coli* OP50. We then scaffolded the assembly using a reference-guided approach as implemented in the software RagTag (version 2.1.0) (Alonge et al., 2022), which took the chromosome-scale assembly for the *P. pacificus* strain PS312 (version El Paco) as reference (Rödelsperger et al., 2017). This resulted in a pseudochromosomal assembly of 11 scaffolds spanning 171 Mb with an N50 value of 26.3 Mb. BUSCO completeness of the genome assembly was estimated to be 92.4% (91.1% single copy + 1.3% duplicates). To annotate protein-coding genes, we employed the PPCAC pipeline (version 1) (Rödelsperger, 2021), which generated evidence-based gene annotations from existing gene annotations for the *P. pacificus* reference strain PS312 (El Paco gene annotations, version 3) (Athanasouli et al., 2020), as well as from RNA-seq data that was generated previously (Rödelsperger et al., 2018). We refined the gene annotations by removing species-specific orphan genes that overlapped in the antisense direction with conserved genes, as we previously observed that coding potential on the antisense strand can result in gene annotation errors (Athanasouli et al., 2020). Evaluating the completeness of the final gene annotations using BUSCO (version 3.1, nematode odb9 data set) revealed a completeness level of 94.9%. The most notable distinguishing feature between the PS1843 and the PS312 genome is a difference of ~16Mb in genome size (Table 1). Whole genome alignments by the minimap2 software (Li, 2018) revealed megabase-sized regions on ChrIII, ChrIV, and ChrX that have no correspondence in the PS312 genome and explain the majority of the genome size difference (Figure 1). These three regions account for roughly 11Mb and contain around 1100 genes, most of which are conserved with other *Pristionchus* species, suggesting that these regions represent ancestral haplotypes that have been retained in the PS1843 genome but were lost in the lineage leading to the PS312 strain.

**Figure 1:**
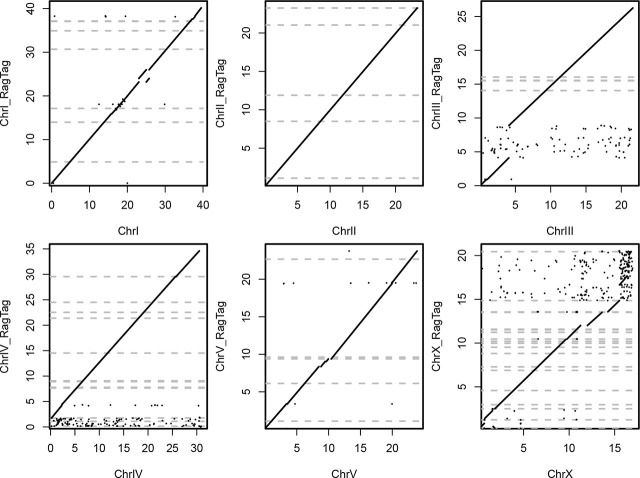
Comparison between the two chromosome-scale *P. pacificus* genomes. The dotplots visualize the megabase coordinates of alignments between the *P. pacificus* PS312 chromosomes (x-axis) and their counterparts in the PS1843 genome (y-axis). The whole genome alignments were generated by the minimap 2 aligner (options: -N 0 -t 6 -x asm5), and the coordinates were extracted from the resulting PAF file. The gray dashed lines mark 52 assembly gaps in the PS1843 genome that were filled with undetermined sequence stretches of 100 nucleotides in length.

**Table 1.** Characteristics of different *P. pacificus* genome assemblies. BUSCO values include single copy and duplicated genes.

	**PS312 (Version El Paco)**	**PS1843 (Prabh and Rödelsperger 2022)**	**PS1843 (This study)**
Number of contigs/scaffolds	47	39,807	11
Total genome size (Mb)	155.0	174.1	171.2
N50 (Mb)	23.9	0.4	26.3
BUSCO (genome) (%)	92.6	88.8	92.4
Number of genes	28,996	26,757	30,098
BUSCO (proteins) (%)	97.6	88.8	94.9

The raw sequencing data and the PS1843 genome assembly have been uploaded to the European nucleotide archive under the study accession PRJEB77003. The data are also available on the pristionchus.org webserver. We hope that the improved PS1843 genome will be useful for future forward genetic studies and evolutionary genomic comparisons at the intra-species level.

## References

[j_jofnem-2024-0063_ref_001] Alonge M., Lebeigle L., Kirsche M., Jenike K., Ou S., Aganezov S., Wang X., Lippman Z. B., Schatz M. C., Soyk S. (2022). Automated assembly scaffolding using RagTag elevates a new tomato system for high-throughput genome editing. Genome Biol.

[j_jofnem-2024-0063_ref_002] Athanasouli M., Witte H., Weiler C., Loschko T., Eberhardt G., Sommer R. J., Rödelsperger C. (2020). Comparative genomics and community curation further improve gene annotations in the nematode Pristionchus pacificus. BMC Genomics.

[j_jofnem-2024-0063_ref_003] Dardiry M., Eberhardt G., Witte H., Rödelsperger C., Lightfoot J. W., Sommer R. J. (2023). Divergent combinations of cis-regulatory elements control the evolution of phenotypic plasticity. PLoS Biol.

[j_jofnem-2024-0063_ref_004] Dieterich C., Clifton S. W., Schuster L. N., Chinwalla A., Delehaunty K., Dinkelacker I., Fulton L., Fulton R., Godfrey J., Minx P., Mitreva M., Roeseler W. (2008). The Pristionchus pacificus genome provides a unique perspective on nematode lifestyle and parasitism. Nat Genet.

[j_jofnem-2024-0063_ref_005] Han Z., Lo W.-S., Lightfoot J. W., Witte H., Sun S., Sommer R. J. (2020). Improving transgenesis efficiency and CRISPR-associated tools through codon optimization and native intron addition in nematodes. Genetics.

[j_jofnem-2024-0063_ref_006] Hiraga H., Ishita Y., Chihara T., Okumura M. (2021). Efficient visual screening of CRISPR/Cas9 genome editing in the nematode Pristionchus pacificus. Dev Growth Differ.

[j_jofnem-2024-0063_ref_007] Igreja C., Loschko T., Schäfer A., Sharma D. R., Quiobe S. P., Aloshy E., Witte H., Sommer R. J. (2022). Application of ALFA-Tagging in the nematode model organisms and. Cells.

[j_jofnem-2024-0063_ref_008] Koren S., Walenz B. P., Berlin K., Miller J. R., Bergman N. H., Phillippy A. M. (2017). Canu: scalable and accurate long-read assembly via adaptive k-mer weighting and repeat separation. Genome Res.

[j_jofnem-2024-0063_ref_009] Li H. (2018). Minimap2: pairwise alignment for nucleotide sequences. Bioinformatics.

[j_jofnem-2024-0063_ref_010] Mayer M. G., Rödelsperger C., Witte H., Riebesell M., Sommer R. J. (2015). The orphan gene dauerless regulates dauer development and intraspecific competition in nematodes by copy number variation. PLoS Genet.

[j_jofnem-2024-0063_ref_011] McGaughran A., Rödelsperger C., Grimm D. G., Meyer J. M., Moreno E., Morgan K., Leaver M., Serobyan V., Rakitsch B., Borgwardt K. M., Sommer R. J. (2016). Genomic profiles of diversification and genotype-phenotype association in island nematode lineages. Mol Biol Evol.

[j_jofnem-2024-0063_ref_012] Moreno E., McGaughran A., Rödelsperger C., Zimmer M., Sommer R. J. (2016). Oxygen-induced social behaviours in Pristionchus pacificus have a distinct evolutionary history and genetic regulation from Caenorhabditis elegans. Proc Biol Sci.

[j_jofnem-2024-0063_ref_013] Prabh N., Rödelsperger C. (2022). Multiple Pristionchus pacificus genomes reveal distinct evolutionary dynamics between de novo candidates and duplicated genes. Genome Res.

[j_jofnem-2024-0063_ref_014] Rillo-Bohn R., Adilardi R., Mitros T., Avşaroğlu B., Stevens L., Köhler S., Bayes J., Wang C., Lin S., Baskevitch K. A., Rokhsar D. S., Dernburg A. F. (2021). Analysis of meiosis in Pristionchus pacificus reveals plasticity in homolog pairing and synapsis in the nematode lineage. Elife.

[j_jofnem-2024-0063_ref_015] Rödelsperger C. (2021). The community-curated Pristionchus pacificus genome facilitates automated gene annotation improvement in related nematodes. BMC Genomics.

[j_jofnem-2024-0063_ref_016] Rödelsperger C., Meyer J. M., Prabh N., Lanz C., Bemm F., Sommer R. J. (2017). Single-molecule sequencing reveals the chromosome-scale genomic architecture of the nematode model organism Pristionchus pacificus. Cell Rep.

[j_jofnem-2024-0063_ref_017] Rödelsperger C., Neher R. A., Weller A. M., Eberhardt G., Witte H., Mayer W. E., Dieterich C., Sommer R. J. (2014). Characterization of genetic diversity in the nematode Pristionchus pacificus from population-scale resequencing data. Genetics.

[j_jofnem-2024-0063_ref_018] Rödelsperger C., Röseler W., Prabh N., Yoshida K., Weiler C., Herrmann M., Sommer R. J. (2018). Phylotranscriptomics of Pristionchus nematodes reveals parallel gene loss in six hermaphroditic lineages. Curr Biol.

[j_jofnem-2024-0063_ref_019] Sieriebriennikov B., Sun S., Lightfoot J. W., Witte H., Moreno E., Rödelsperger C., Sommer R. J. (2020). Conserved nuclear hormone receptors controlling a novel plastic trait target fast-evolving genes expressed in a single cell. PLoS Genet.

[j_jofnem-2024-0063_ref_020] Simão F. A., Waterhouse R. M., Ioannidis P., Kriventseva E. V., Zdobnov E. M. (2015). BUSCO: assessing genome assembly and annotation completeness with single-copy orthologs. Bioinformatics.

[j_jofnem-2024-0063_ref_021] Sommer R. J., Dardiry M., Lenuzzi M., Namdeo S., Renahan T., Sieriebriennikov B., Werner M. S. (2017). The genetics of phenotypic plasticity in nematode feeding structures. Open Biol.

[j_jofnem-2024-0063_ref_022] Sommer R. J., McGaughran A. (2013). The nematode Pristionchus pacificus as a model system for integrative studies in evolutionary biology. Mol Ecol.

[j_jofnem-2024-0063_ref_023] Srinivasan J., Sinz W., Lanz C., Brand A., Nandakumar R., Raddatz G., Witte H., Keller H., Kipping I., Pires-daSilva A., Jesse T., Millare J. (2002). A bacterial artificial chromosome-based genetic linkage map of the nematode Pristionchus pacificus. Genetics.

